# Sarcoidosis and Monoclonal Gammopathy of Undetermined Significance (MGUS): A True Association or Just a Coincidence?

**DOI:** 10.1155/2018/3790760

**Published:** 2018-09-25

**Authors:** Mohamed Hassanein, Lilit Karapetyan, Afshan Khan, Supratik Rayamajhi

**Affiliations:** Michigan State University, Clinical Center, 788 Service Road, East Lansing, MI 48824, USA

## Abstract

Sarcoidosis is a systemic inflammatory disease characterized by the presence of noncaseating granulomas in different organs. Sarcoidosis associated with monoclonal gammopathy of undetermined significance (MGUS) is a rare finding with only 10 cases reported to date. We describe a 79-year-old male patient who presented with dry mouth for 4 months. Lip biopsy done prior to admission showed nonnecrotizing epithelioid cell granulomas. On admission, laboratory analysis was significant for elevated calcium, decreased parathyroid hormone, increased erythrocyte sedimentation rate, undetectable parathyroid hormone-related peptide (PTHrp), mildly decreased 25-hydroxyvitamin D, elevated 1,25-dihydroxyvitamin D, elevated angiotensin converting enzyme, and positive Bence Jones protein in the urine. Serum protein electrophoresis showed an elevated gamma globulin level at 38% and an IgG monoclonal gammopathy with an M-spike of 1.47. Bone marrow biopsy was consistent with MGUS. The patient showed significant improvement with steroids and was discharged with close follow-up from nephrology and oncology. Salivary gland involvement in patients with sarcoidosis is a rare finding. Our case is a valuable addition to the small number of cases described in the literature supporting an association between plasma cell disorders and sarcoidosis. Larger prospective studies are needed to determine if a true association between the two diseases exists.

## 1. Introduction

Sarcoidosis is a systemic inflammatory disease characterized by the presence of noncaseating granulomas in different organs. The most common affected sites include the lungs, skin, and lymph nodes. Patients may be asymptomatic or may have a wide variety of clinical manifestations depending on the organs involved. Persistent cough, generalized weakness, fever, night sweats, peripheral lymphadenopathy, erythema nodosum, fatigue, and incidental chest radiograph abnormalities are the most common disease manifestations. The nonspecific clinical picture of sarcoidosis often leads to a late diagnosis of the disease [1].

An association between sarcoidosis and malignant neoplasms has been described in the literature [2]. Sarcoidosis-lymphoma syndrome is characterized by the development of lymphoproliferative disorders following the diagnosis of sarcoidosis, with Hodgkin's lymphoma being the most common type [3, 4]. Sarcoidosis association with monoclonal gammopathy of undetermined significance (MGUS) is a rare finding with only 10 cases reported until date [5–13].

We describe a patient who presented with dry mouth and was later found to have concurrent sarcoidosis and MGUS.

## 2. Case Presentation

A 79-year-old male with a past medical history of hypothyroidism and benign prostatic hyperplasia presented with dry mouth for four months. Family history was significant for the presence of Crohn's disease and systemic lupus erythematosus (SLE) in his sister. He was initially referred to an oral surgeon who performed a lip biopsy two weeks prior to admission revealing nonnecrotizing epithelioid cell granulomas. The patient was prescribed a mouthwash solution containing diphenhydramine, nystatin, lidocaine, hydrocortisone, and tetracycline. He was referred to a rheumatologist. His xerostomia significantly worsened prior to his appointment with rheumatology so he decided to go to the emergency department (ED). He presented to the ED with generalized weakness and decreased oral intake secondary to mouth pain resulting in a 30-pound weight loss over four months. Physical exam was remarkable for dry oropharyngeal mucosa. Laboratory analysis revealed an elevated serum calcium of 12.71 mg/dL (reference range: 8.4–10.7 mg/dL), an increased ionized calcium of 1.9 mmoL/L (reference range: 1.10–1.30 mmoL/L), an increased creatinine level of 3.81 mg/dL (reference range: 0.7–1.3 mg/dL), a decreased parathyroid hormone level of 6.5 pg/mL (reference range: 15–65 pg/mL), and an increased erythrocyte sedimentation rate (ESR) of 55 mm/hr (reference range: 0–15 mm/hr). Despite adequate fluid hydration, his calcium level remained elevated. Further workup for hypercalcemia revealed undetectable parathyroid hormone-related peptide (PTHrp), mildly decreased 25-hydroxyvitamin D at 18.1 ng/ml (reference range: 20–100 ng/ml), and an elevated 1,25-dihydroxyvitamin D at 72 pg/ml (reference range: 18–64 pg/ml).

Further workup for hypercalcemia showed an elevated angiotensin-converting enzyme at 91 U/L (reference range: 18–53 U/L). Bence Jones protein revealed free lambda light chains in the urine. Serum protein electrophoresis showed an elevated gamma globulin level of 38% and an IgG monoclonal gammopathy with an M-spike of 1.47. Immunoglobulin free light chain revealed elevated kappa free light chains at 5.79 and elevated lambda free light chains at 14.1 with a kappa to lambda ratio of 0.4. A bone marrow biopsy was done and was sent for pathology.

Further workup for dry mouth showed positive antinuclear antibody (ANA) at a titer of 1 : 160, and the other rheumatologic workup was negative.

**Figure 1:**
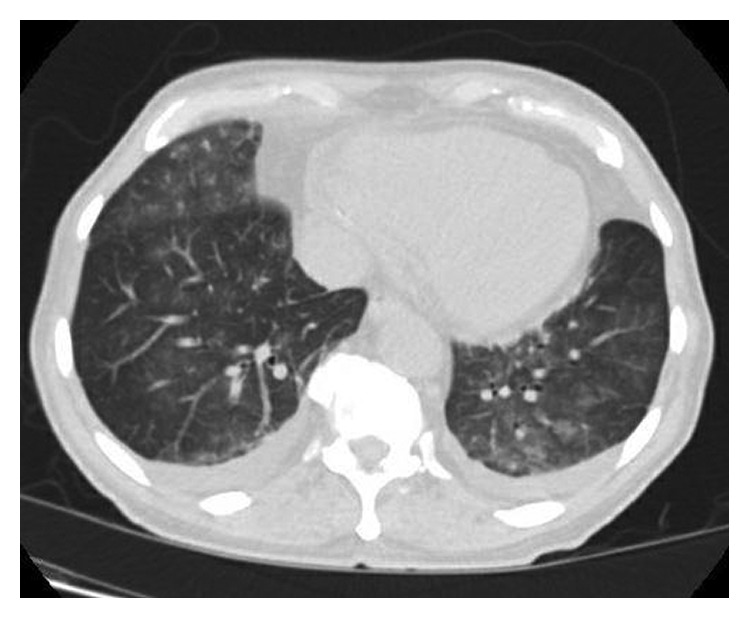
High-resolution CT showing bilateral ground glass opacities and bilateral pleural effusion.

The constellation of findings prompted further workup for sarcoidosis. Chest radiograph (CXR) showed minimal hilar lymphadenopathy which was more pronounced on the right side, bibasilar infiltrates and mild bilateral pleural effusions. High-resolution computerized tomography (HRCT) of the chest showed prominent hilar densities, ground glass opacities, and bilateral pleural effusions (Figure 1). He was started on prednisone 40 mg/day and noticed immediate symptomatic improvement. His calcium level normalized, and his acute kidney injury resolved. Bone marrow biopsy results revealed lambda-restricted plasma cell neoplasm with 5%–6% of bone marrow cellularity consistent with MGUS. The patient was discharged with a one-month follow-up from nephrology and oncology.

## 3. Discussion

Sarcoidosis is a chronic inflammatory disease of unknown etiology. It is thought to occur secondary to exaggerated immunologic response to environmental factors in genetic susceptible patients. The diagnosis is made by clinical and radiological criteria, histopathological finding of noncaseating granulomas, and absence of alternative diagnoses. The pathophysiology involves activation of different arms of the immune system including lymphocytes, macrophages, and antigen-presenting cells [14]. Upon exposure to different antigens, antigen-presenting cells produce tissue necrosis factors alpha (TNF-α) and several interleukins that stimulate cluster of differentiation 4 (CD4) T cells to differentiate into T1 helper- (TH1-) and T2 helper- (TH2-) like cells, which secrete various interleukins and interferon gamma, leading to the formation of granulomas [15].

The presentation of sarcoidosis in our patient was atypical in several ways. Sarcoidosis most commonly affects patients between 20–40 years of age. Persistent cough, generalized weakness, fever, night sweats, peripheral lymphadenopathy, erythema nodosum, fatigue, and incidental chest radiograph abnormalities are the most common disease manifestations. Our patient was 79 years old and presented with dry mouth, both of which are atypical for sarcoidosis. Orofacial sarcoidosis is a rare finding. Common orofacial symptoms of sarcoidosis include pain, nasal obstruction, loosening of the teeth, swelling of the mandible, and localized swelling and ulcers of the oral cavity. A few case series reported oral involvement as an initial presentation of the disease [16–18]. In addition, a case-control study by Baughman et al. reported a 3.9% involvement of the salivary glands in 736 patients with biopsy-proven sarcoidosis [19].

Sarcoidosis is divided into 5 stages based on CXR findings (Table 1) [20]. In our case, the minimal hilar lymphadenopathy with bilateral infiltrates and pleural effusions on CXR is nonspecific for sarcoidosis. HRCT is used to confirm CXR findings and can detect parenchymal abnormalities not evident on CXR. The most common radiologic findings of sarcoidosis on HRCT include lymphadenopathy, perilymphatic micronodules, interstitial thickening, and pulmonary fibrosis [20, 21]. Bilateral ground glass opacities are a rare finding of sarcoidosis. In our case, the presence of bilateral ground glass opacities with prominent hilar densities is atypical for sarcoidosis. Pleural effusion in sarcoidosis is also rare and most commonly occurs in stages 1 and 2 [22]. Without thoracentesis and thoracoscopy with pleural biopsy, a definite diagnosis of pleural sarcoidosis cannot be made. In our case, the pleural fluid was minimal and insufficient for thoracentesis, posing a risk of pneumothorax. The diagnosis was strongly supported by the patient's presentation of dry mouth, elevated calcium, 1,25-dihydroxyvitamin D, ACE, the pathological finding of noncaseating granulomas on lip biopsy, and the drastic improvement on steroids. One positive biopsy result is usually sufficient for diagnosis. An elevated ACE level is present in approximately 60% of patients at the time of diagnosis, but it lacks both sensitivity and specificity. Inflammatory biomarkers similar to interleukin-2 receptor, neopterin, chitotriosidase, lysozyme, KL-6, and amyloid A might be a better option to assess disease activity. Positive ANA is detected in 29% of patients with sarcoidosis. Hypercalciuria is detected in 40% of patients, and hypercalcemia is seen in 11% of patients. Hypercalcemia can lead to intrarenal calcium deposition and renal failure [1, 15, 23].

Patients with sarcoidosis have an increased risk for developing cancer of the lung, stomach, small intestine, liver, and lymphoid tissues. The relative risk of developing lymphoid malignancies was found to be 13.2% in patients with sarcoidosis [24, 25].

The simultaneous occurrence of sarcoidosis and plasma cell disorders is a rare finding. To the best of our knowledge, only 10 cases have been reported thus far (Table 2). The cases described in the literature show a median latency period of four years between the diagnosis of sarcoidosis and MGUS. In 30% of cases, sarcoidosis was the initial diagnosis, and in another 30%, MGUS was the initial diagnosis. Five cases, including ours, report simultaneous presentation of MGUS and sarcoidosis at the same time. The median age of onset in these patients was 61, and 70% were female. 80% had elevated IgG monoclonal protein, as was seen in our patient.

**Figure 2:**
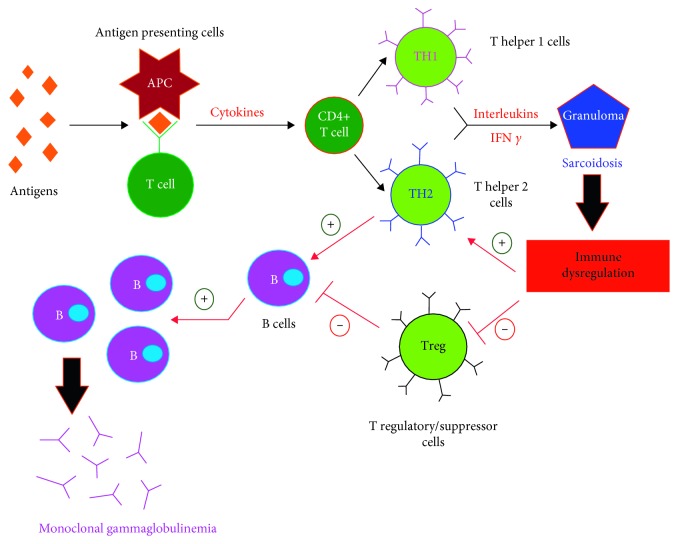
Proposed pathogenesis of MGUS in patients with sarcoidosis. Abbreviations: APC (Antigen-presenting cells), T cell (T lymphocytes), CD4+ T cell (cluster differentiation 4 positive T cell), TH1 (T Helper 1 cells), TH2 (T Helper 2 cells), IFN *γ* (Interferon gamma), Treg (Regulatory T cells), B Cells (B lymphocytes).

There are no epidemiologic data to our knowledge to support the increased risk of plasma cell disorders in patients with sarcoidosis. It is suggested that dysregulation of the immune system may induce development of autonomous plasma cell clones and result in the uncontrolled production of the monoclonal immunoglobulins. Activation of CD4 positive T helper cells, suppression of CD8 positive T regulatory/suppressor cells, and increased cytokine production may contribute to the continuous stimulation of B cells, causing monoclonal or polyclonal hypergammaglobulinemia (Figure 2) [8]. This hypothesis was supported by Hunninghake and Crystal, who found increased numbers of IgG- and IgM-secreting cells in the blood in untreated patients with pulmonary sarcoidosis, compared to normal individuals [26]. Moreover, they extracted T lymphocytes from the lungs of patients with high and low proportions of T lymphocytes and cocultured each of the T lymphocyte samples with blood mononuclear cells of normal individuals. T lymphocytes from the group with high proportion of T lymphocytes induced differentiation of the cocultured mononuclear cells into immunoglobulin-secreting cells. On the other hand, the diagnosis of MGUS in patients with sarcoidosis could be a coincidence. Dispenzieri et al. studied the prevalence of MGUS in a retrospective population-based cohort of 18,357 patients. The prevalence of MGUS in 79-year-old males was found to be approximately 7% [27]. Therefore, the occurrence of MGUS in our 79-year-old gentleman could have occurred by chance.

The annual risk of progression of MGUS to multiple myeloma (MM) is approximately 1% in general population. Up to 16% of patients with MGUS may develop MM after >30 years. Risk factors for progression per Mayo Criteria include an M-protein level >1.5 g/dL, Non-IgG isotype (IgA or IgM), and an abnormal Kappa-Lambda free light chain ratio (<0.26 or >1.65). Monitoring for progression to MM depends on the number of risk factors present. Patients with no risk factors are recommended to repeat serum protein electrophoresis (SPEP) in 6 months and every 2-3 years thereafter. Patients with 1 or more risk factors are recommended to check serum LDH, B2 microglobulin, C-reactive protein, and bone marrow biopsy at time of diagnosis, in addition to a CT abdomen for patients with IgM subtype to rule out Waldenström's macroglobulinemia. If all tests are unremarkable, these patients should monitor complete blood count (CBC), SPEP and creatinine in 6 months and then, yearly for life [28]. Patients with MGUS and sarcoidosis might be associated with an increased risk of development of MM. Indeed, in patients with sarcoidosis, MGUS is thought to progress to MM more rapidly than in the general population [8]. We suggest monitoring these patients with CBC, SPEP, and creatinine 6 months after diagnosis and annually for life similar to those with the risk factors mentioned previously to early detect and possibly prevent complications of progression to MM.

## 4. Conclusion

In conclusion, we describe a patient who presented with dry mouth and was diagnosed simultaneously with MGUS and sarcoidosis. Salivary gland involvement in patients with sarcoidosis is a rare finding. Our patient was treated with steroids which resulted in symptomatic improvement. Our case is one of few others in the literature describing concurrent plasma cell disorders and sarcoidosis. Larger prospective studies are needed to determine if an association between the two diseases exists and define the nature of association and whether causality exists.

## Figures and Tables

**Table 1:** Stages of Sarcoidosis according to chest X-ray findings.

Stage	Chest X-ray findings
0	Normal chest X-ray
1	Mediastinal lymphadenopathy
2	Mediastinal lymphadenopathy with parenchymal lesions
3	Parenchymal lesions only
4	Pulmonary fibrosis

**Table 2:** Cases with sarcoidosis and MGUS reported in the literature.

Author	Year, country	Age/sex	Ethnicity/race	First Dx (Sarcoidosis/MGUS)?	Latency	Monoclonal protein type	Progression to MM?	Prognosis/outcome
Antela Lopez [5]	1992, Spain	68 F	UNK	Sarcoidosis	3 YRS	IgM	UNK	Treated with prednisone, diagnosed with NHL 3 YRS later, treated with chemotherapy ⟶ complete remission
Sharma et al. [6]	1992, USA	53 F	AA	Sarcoidosis	17 YRS	IgG	N	Stable 16 YRS later
Sharma et al. [6]	1992, USA	69 F	Caucasian	Diagnosed same time	UNK	IgG	N	UNK
Berner et al [7]	1999, Germany	67 M	UNK	Diagnosed same time. Diagnosed with renal sarcoidosis	UNK	IgG	N	Normalization of kidney function with prednisolone therapy
Sen et al [8]	2002, USA	49 F	AA	Diagnosed same time	UNK	IgG	Y (4 YRS)	Stable 30 MS later
Saad et al. [9]	2009, Israel	67 F	ME/Egyptian	Sarcoidosis	3 YRS	IgG	UNK	UNK
Ghafour and Almakki [10]	2014, USA	69 M	UNK	MGUS. Later diagnosed with renal sarcoidosis	1 YRS	IgG	N	UNK
Guboutan et al. [11]	2016, USA	57 F	Caucasian	MGUS	4 YRS	UNK	N	Treated with steroid taper for 4 MS. Resolution of symptoms 2 YRS later
Abdulai and Englert [12]	2016, USA	58 M	Caucasian	MGUS. Later diagnosed with neurosarcoidosis	2 MS	IgG	N	UNK
Armaral et al. [13]	2017, Brazil	54 F	UNK	Diagnosed same time	UNK	IgG	N	UNK

Abbreviations: M: male; F: female; AA: African American; ME: Middle Eastern; UNK: unknown; not mentioned. Y: yes; N: no; YRS: years; MS: months; NHL: non-Hodgkin's lymphoma. MGUS: Monoclonal gammopathy of undetermined significance.
